# Mirror exposure in adolescents with anorexia nervosa: a feasibility study on body image disturbance and attentional bias

**DOI:** 10.1007/s40519-026-01880-2

**Published:** 2026-06-08

**Authors:** Maarit Pelzer, Jessica Werthmann, Jennifer Svaldi, Christian Fleischhaker, Barbara Haack-Dees, Fritz Renner, Brunna Tuschen-Caffier

**Affiliations:** 1 Department of Psychology, Clinical Psychology and Psychotherapy, Albert-Ludwigs-University of Freiburg, Freiburg, Germany; 2 Faculty of Science, Clinical Psychology and Psychotherapy, Eberhard Karls University Tübingen, Tübingen, Germany; 3 Department of Child and Adolescent Psychiatry, Psychotherapy and Psychosomatics, Freiburg University Hospital, Freiburg, Germany

**Keywords:** Anorexia nervosa, Adolescents, Body image disturbance, Mirror exposure, Attentional bias

## Abstract

**Purpose:**

Body image disturbances (BID) are central to anorexia nervosa (AN), influencing its development, maintenance and relapse. While mirror exposure (ME) has been shown to reduce BID in adults, research in adolescents is limited. This pilot randomized controlled trial examined the feasibility and effects of ME, added to treatment as usual (TAU), in adolescent inpatients with AN. In addition, we explored changes in body-related attentional bias as a potential mechanism of change.

**Methods:**

Adolescent female inpatients with AN were randomized to receive either 12 ME sessions over 4 weeks (ME group, *n* = 11) or TAU (*n* = 13) in a pre-post design. Primary outcomes were global measures of BID and eating disorder (ED) pathology. Attentional bias was assessed using two eye-tracking paradigms.

**Results:**

Relative to TAU, ME did not improve BID or ED pathology. Both groups showed reductions in BID over time. Exploratory post-hoc analyses indicated reductions in body control and avoidance behaviors in the ME but not the TAU group. An attentional bias toward self-defined unattractive body parts was present but did not change over time in either group.

**Discussion:**

In this pilot trial, ME did not provide additional benefits over TAU in improving global BID or ED pathology in adolescent inpatients with AN. Improvements in BID may reflect general therapeutic effects of inpatient treatment. Exploratory analyses indicated potential effects on specific body-related behaviors but require cautious interpretation due to limited power. Future research should examine for whom and at what stage body image–focused interventions may be beneficial.

**Level of evidence:**

Level I, randomized controlled trial.

**Trial registration:**

The study is registered on the German Clinical Trial Register (DRKS; registration number: DRKS0019104).

**Supplementary Information:**

The online version contains supplementary material available at 10.1007/s40519-026-01880-2.

## Introduction

Anorexia nervosa (AN) is a serious mental disorder associated with multiple physical complications and high mortality, with adolescence as typical age of onset [1]. Longitudinal data indicate low remission rates and considerable relapse risk, underscoring the need for interventions targeting core maintaining factors such as body image disturbance (BID) [2].

Mirror exposure (ME) is a promising approach to reduce BID. Evidence from systematic reviews and meta-analyses supports beneficial effects of exposure-based body image interventions in eating disorders (EDs; [3, 4]); however, most studies have focused on adults or on multimodal programs in which the specific contribution of ME remains unclear, and empirical data on ME in adolescents with AN are scarce [5]. Similarly, evidence regarding the mechanisms underlying ME remains limited and mixed [4].

To address this gap, we conducted a pilot randomized controlled trial to examine the feasibility and preliminary effects of an intensive ME intervention (12 sessions over 4 weeks) on BID, ED pathology, and attentional bias toward negatively evaluated body areas—changes that have been postulated as a potential mechanism of action—in adolescents with AN receiving inpatient treatment. Given the central role of behavioral and weight-related interventions in acute AN treatment, it remains unclear whether additional body image–focused interventions provide incremental benefits in this context. We hypothesized that ME would lead to greater reductions in global BID ( body dissatisfaction, body checking, and avoidance) and ED pathology compared to treatment-as-usual (TAU). We further examined associations between changes in BID, ED pathology, and attentional bias, as well as exploratory session-based changes in body evaluation and affect and potential predictors of treatment response.

## Methods

The study was approved by the ethics committee of the Albert-Ludwigs University Freiburg (545/17) and preregistered in the German Clinical Trials Register (DRKS00019104). A study protocol has been published (see [6]).

### Participants

Patients with AN were recruited via the Department of Child and Adolescent Psychiatry at the University Medical Center Freiburg. Inclusion criteria were an International Classification of Diseases (10th edition, ICD) diagnosis of AN or atypical AN; participants had to have reached a BMI-age-percentile of ≥ 10th at study start to ensure sufficient cognitive and medical stability for participation, and age 12–21 years. Associated problems (e.g., depression, self-esteem) were assessed to further characterize the sample. Exclusion criteria included ongoing tube feeding, current psychotic or bipolar disorders, recent substance dependence, high suicide risk, severe medical conditions, pregnancy, or lactation. Recruitment occurred from 2018 to 2023 with a target sample of *N* = 42. Of 63 individuals contacted, 28 consented. The relatively low consent rate may reflect the clinical burden of the population and the demands of the intervention. Given the reduced sample size, this study is considered a pilot investigation. The participant flow chart is provided in the Supplementary Material. A 3-month follow-up assessment was planned; however, due to low response rates (41%), follow-up data were not included in the present analyses.

### Material

An overview of assessment instruments is provided in Table 1. Detailed descriptions of all outcome measures, procedures and the intervention can be found in the published protocol [6].

**Table 1:** Overview of assessment instruments

Constructs	Assessment
Description of the sample	
Sample characteristics	Age, BMI, BMI-percentile and illness duration
Depression severity	Beck Depression Inventory (BDI-II; [7]; German version: [8])
Self-esteem	Rosenberg Self-Esteem Scale (RSE; [9]; German version: [10])
Outcome variables	
Components of BID	
Affective and cognitive components	Body Shape Questionnaire (BSQ, [11]; German version: Fragebogen zum Figurbewusstsein, FFB; [12])
Behavioral components	Body Checking Questionnaire (BCQ; [13]; German version: [14])
	Body Image Avoidance Questionnaire (BIAQ; [15], German version: [16])
ED pathology	Child Eating Disorder Examination Questionnaire (ChEDE-Q; [17]; German version: [18])
Affect reactivity over time	German version of the Positive and Negative Affect Scale (PANAS; [19])
Body evaluation over time	“Body Questionnaire” (e.g. as [20]) to indicate which body parts they considered most attractive/unattractive body part on a scale from 1 (attractive) to 10 (unattractive)

*BCQ* Body Checking Questionnaire, *BDI* Becks Depression Inventory, *BIAQ* Body Image Avoidance Questionnaire, *BMI* Body mass index, *BSQ* Body Shape Questionnaire, *FFB* Fragebogen zum Figurbewusstsein, *PANAS* Positive and Negative Affect Scale, *ChEDE-Q* Child Eating Disorder Examination Questionnaire, *RSE* Rosenberg Self-Esteem Scale

### Attentional bias

Body-related attentional biases were assessed with two eye-tracking paradigms, using iViewXTM2 software.

In the exogenous cueing task, participants viewed images of their own body (self-body, SB) or a BMI-matched control body (CB) for 3000 ms before responding to a cue. Bias indices were fixation duration and frequency toward to self-defined attractive and unattractive body areas.

In the dot-probe task, stimulus pairs (SB/CB, SB/vase and CB/vase) were shown for 3000 ms, followed by a cue. Bias was indexed as direction bias to SB or CB compared to competing stimuli.

### Intervention

All participants received inpatient TAU, which focused on weight restoration and normalization of eating behavior and included regular individual psychotherapy sessions; further details are provided in the study protocol [6]. No structured body image–focused interventions were provided during the study period. Following randomization, participants either continued with TAU alone or received TAU plus 12 ME sessions (60 min./over 4 weeks), following a standardized protocol. ME was delivered by trained post-graduate psychologists under supervision by an experienced licensed therapist (BTC).

### Data processing

Eye-movement data were analyzed using BeGaze (SMI). Regarding the exogenous cueing task, fixation durations were computed for individually defined areas of interest (AOIs), corresponding with the three self-perceived most “attractive” and “unattractive” body parts of SB and CB. Durations were averaged across trials and normalized by mean fixation time on the entire body, yielding a relative duration index that reflects attentional bias toward personally salient areas; higher values indicate a stronger duration bias toward the selected AOIs. Relative fixation frequency was calculated by dividing the number of fixations within these AOIs by the total number of trials in which an AOI fixation occurred; higher values reflect stronger attention allocation to these areas.

For the dot-probe task, the position of the first fixation within each stimulus pair (SB/CB, SB/vase, CB/vase) was recorded. Direction bias scores were calculated by dividing the number of first fixations on one stimulus by the total possible first-fixation opportunities within that pair. Scores above 0.50 indicate a stronger orientation toward SB in the SB—CB and SB—vase comparisons, and toward CB in the CB—vase comparison.

### Statistical analysis

Assumptions were checked and, when necessary, Greenhouse–Geisser correction was applied. Significance level was set at *p* < 0.05. Partial eta squared (η^2^_p_) was reported for analyses of variance (ANOVA) and Cohen’s d for between-group comparisons. Spearman’s ρ was used for correlation analysis. Independent t-tests compared the ME and TAU group on sample characteristics.

#### Primary research question: impact of ME on BID and ED pathology

A mixed-design multivariate analysis of variance (MANOVA) assessed group (ME vs. TAU) ×  time (pre/post) effects on BID measures. In addition, a 2 × 2 mixed ANOVA, with Group and Time as factors, tested changes in ED pathology. Correlations examined associations between changes in BID and ED pathology.

#### Secondary research question: impact of ME on body-related attentional bias

Baseline attentional bias was tested with paired-samples t-tests on duration bias for “unattractive” and “attractive” parts of the SB versus the CB. Two 2 × 2 × 2 × 2 mixed ANOVAs were then conducted on duration bias and relative fixation frequency, with Group (ME vs. TAU) as between-subject factor, and Time (pre/post), Evaluation (“attractive” vs. “unattractive”) and Body (SB vs. CB), as within-subject factors.

Three separate 2 × 2 mixed ANOVAs were conducted for each comparison (SB vs. CB, SB vs. vase, CB vs. vase), with Group and Time as factors and direction bias as dependent variables.

Associations between changes in the attentional bias scores and changes in body dissatisfaction, body checking and avoidance were examined by correlational analyses.

#### Exploratory analysis

Changes in affect and body evaluation across sessions were compared between ME and TAU using repeated-measures ANOVAs. Potential predictors of change in BID were first examined in bivariate correlations and then entered in post-hoc Huber regressions. Additional post-hoc ANOVAs examined Group × Time effects on BID components and ED pathology at scale and item level. Finally, Huber regressions in a pooled sample (including TAU participants who later received ME), tested whether baseline attentional bias predicted symptom change.

Given the pilot nature of the study, the limited sample size, and the number of analyses conducted, results—particularly from exploratory and post-hoc analyses—should be interpreted with caution.

## Results

### Sample characteristics

Diagnoses included restrictive-type AN (*n* = 19), purging-type AN (*n* = 4), and atypical AN (*n* = 1). Comorbid diagnoses were social phobia (*n* = 14), depressive episodes (*n* = 6), and generalized anxiety disorder (*n* = 4); the profile of comorbid diagnoses is comparable to research in this filed [21]. Groups did not differ in age, BMI percentile, or duration of inpatient treatment (see Table 2).

**Table 2:** Sample characteristics and baseline comparison

	ME	TAU			
	*n* = *11*	*n* = *13*	*t*(22)	*p*	Cohen’s* d*
Age (years), *M (SD)*	14.00 (1.67)	15.07 (1.04)	1.93	0.067	0.79
BMI percentile, *M (SD)*	17.70 (6.96)	21.99 (11.53)	1.05	0.303	0.43
Duration of treatment (in days), *M (SD)*	85.73 (42.81)	73.62 (24.01)	-0.87	0.392	-0.36

*BMI* Body Mass Index, *ME* Mirror Exposure group, *TAU* Treatment As Usual group. Mean parameter values are shown for the ME group (*n* = 11) and the TAU group (*n* = 13)

Two participants were included with BMIs slightly below the predefined cutoff but were retained because their values were not outliers.

### Impact of ME on BID and ED pathology

Against expectation the MANOVA on BID measures revealed no significant Group x Time interaction, *F*(1, 22) = 0.87, *p* = 0.361, *η*^2^_p_ = 0.038, Wilks’ Λ = 0.962, but a significant main effect of Time, *F*(1, 22) = 284.06, *p* < 0.001, *η*^2^_p_ = 0.928, Wilks’ Λ = 0.072, indicating improvements over time across groups (means and standard deviations are provided in the Supplementary Material).

Similarly, the ANOVA on ED pathology showed no statistically significant interaction between Group and Time, *F*(1, 22) = 0.979, *p* = 0.333, *η*^2^_p_ = 0.043.

Changes in BID components correlated strongly with changes in ED pathology (e.g., *r*ₛ = 0.81 and *r*ₛ = 0.77, both *p* < 0.01), with comparable associations in both groups.

### Body-related attentional bias as potential mechanism

#### Exogenous cueing paradigm

As expected, at pre-test, participants showed a duration bias toward “unattractive” areas of their own body, with significantly longer fixation on these areas compared to the “unattractive” regions on a control body (SB vs. CB; *t*(22) = 3.04, *p* = 0.006). No such bias was observed for “attractive” areas, *t*(22) = -1.38, *p* = 0.182.

Results of the 2 × 2 × 2 × 2 ANOVA’s are summarized in Table 3. A significant four-way interaction was observed for duration bias: the ME group showed an increase in bias toward the most attractive CB areas, whereas the TAU group showed an increase in bias toward the most unattractive CB areas, with minimal changes for the SB in both groups. This finding should be interpreted with caution due to assumption violations. Three-way interactions for duration bias and all effects for relative fixation frequency were nonsignificant.

**Table 3:** Results of 2 × 2 × 2 × 2 ANOVAs for duration bias and relative fixation frequency

Measure	Interaction	*F*(1, 21)	*p*	*η*^*2*^*ₚ*
Duration bias	Group × Time × Evaluation × Body	10.38	0.004	0.33
	Group × Time × Evaluation	0.86	0.364	0.04
	Group × Time × Body	0.07	0.793	0.00
Relative fixation frequency	Group × Time × Evaluation × Body	0.78	0.386	0.04
	Group × Time × Evaluation	0.08	0.934	0.00
	Group × Time × Body	0.00	0.965	0.00

Body = self body vs. control body, Evaluation = “attractive” vs. “unattractive”, Group = mirror exposure group vs. treatment as usual group, Time = pre vs. post, *η*^2^ₚ = partial eta squared

#### Dot probe paradigm

In the SB vs. CB comparison, a 2 × 2 mixed ANOVA, as expected revealed a significant Group × Time interaction, *F*(1, 20) = 5.60, *p* = 0.028, *η*^2^_p_ = 0.219, but no main effects (all *Fs* < 1.96, all *ps* > 0.176). A post-hoc *t*-test indicated a significant pre-intervention difference, *t*(20) = –2.09, *p* = 0.05, with stronger direction bias toward SB in the ME group. In contrast, the SB/CB vs. vase comparisons revealed no significant interaction or main effects (all *F*s < 0.57, all *p*s ≥ 0.459, and all *F*s < 0.07, all *p*s ≥ 0.796, respectively).

Correlations between changes in the attentional biases’ coefficients and changes in the BID components or general ED pathology were nonsignificant (all Spearman’s ρ > -0.239, all *p*s > 0.287).

#### Treatment acceptance

Most participants rated ME positively: 91% would recommend it, 82% found it helpful, and 73% were satisfied with session frequency, although all reported that the intervention was challenging.

#### Changes in affect and body evaluation

Against expectation, repeated-measures ANOVAs showed no significant Group × Time interactions or main effects for body evaluation (all *F*s < 1.37, all *p*s ≥ 0.257), and affect (all *F*s < 1.84, all *p*s ≥ 0.124).

### Exploratory analyses

#### Predictors of change

Regarding predictors of change, reductions in body dissatisfaction were positively associated with age (ρ = 0.433, *p* = 0.039) and baseline ED pathology (ρ = 0.430, *p* = 0.036), while no other variables showed significant correlations with changes in the BID components (all Spearman’s ρ > -0.223, *p* > 0.222).

Robust regression models showed greater reductions in body dissatisfaction in both groups (ME, including TAU participants who received ME post-study:* β* = 4.42, *p* = 0.045; TAU, without intervention: *β* = 11.09, *p* = 0.007). An interaction model found no significant Age × Group interaction (*β* = -6.67, *p* = 0.136), indicating comparable age effects across groups.

Baseline ED pathology also predicted change in body dissatisfaction (*β* = 4.72, *p* = 0.037), independent of age. In a combined model, neither predictor interacted with group (*p*s > 0.44), and the effect of age was reduced to trend level (*p* = 0.070). Older age and higher baseline ED pathology were thus associated with greater reductions in body dissatisfaction, regardless of treatment condition.

#### Post-hoc analyses

Given the absence of Group × Time effects on global BID, we additionally conducted exploratory post-hoc analyses at the subscale and item level, as global measures may not capture the specific changes targeted by ME. These analyses revealed several Group × Time interactions in checking-, avoidance-, and weighing-related behaviors, including effects on the BIAQ *grooming and weighing* subscale and on items reflecting mirror checking, checking reflections in surfaces, and self-weighing concerns. Most other analyses showed Time effects only. Baseline attentional bias did not predict symptom change (*p*s > 0.05). Full statistical details are provided in the Supplementary Materials.

## Discussion

This study examined the feasibility and potential effects of ME in adolescents with AN during inpatient treatment, focusing on cognitive, affective, and behavioral components of BID as well as ED pathology, and body-related attentional bias. Contrary to expectations, ME did not provide additional benefits over TAU in improving global BID or ED pathology in adolescent inpatients with AN. All participants showed an attentional bias toward “unattractive” body areas at baseline, which remained unchanged after ME. Body evaluation and affect were also unaffected.

### Limited effects on global outcomes

Limited statistical power likely constrained the detection of group differences, and global BID measures may have been too broad to capture changes associated with a targeted intervention such as ME. Exploratory post-hoc analyses indicated changes in specific checking-, avoidance-, and weighing-related behaviors. However, these findings should be interpreted with caution given the limited sample size and increased risk of type I error. Our null results on global measures align with Sasse et al. [22], while our exploratory findings resemble those by Biney et al. [23]. Although caution is warranted in interpreting findings from the present study, these results are consistent with evidence indicating that specialized AN treatment do not consistently lead to greater improvements in psychological symptoms compared to standard or control treatments [24]. Thus, it remains unclear whether highly specific body image-focused interventions provide incremental benefit beyond inpatient treatment itself. Their potential added value may depend on patient characteristics, treatment timing, or stage of recovery.

### Attentional bias and emotional processes in ME

Baseline attentional bias toward negatively evaluated body areas was in line with previous research, a pattern also found in healthy adolescents with elevated body dissatisfaction [25]. This bias remained unchanged after ME, consistent with prior research reporting limited or inconsistent effects following body exposure in adolescents with AN [23] and women with high body dissatisfaction [26], although reductions have been reported in individuals with binge-eating disorder [27]. More recent ME approaches combining attentional bias modification, including virtual reality, show initial effects, but evidence remains limited to small pilot studies. Another potential amplifier of exposure effects may be emotional engagement, however affective reactivity during ME was not assessed. For future investigations a real-time assessment may help clarify in-session processes.

### Age as potential predictor

Older adolescents benefited more than younger ones, independent of treatment condition. Younger patients may lack the cognitive maturity and reflective capacity to fully benefit from interventions targeting abstract constructs such as body image or self-esteem [28]. While this interpretation remains tentative and requires further research, the pattern suggests that age-appropriate adaptations could improve the intervention’s suitability for younger patients and may be worth considering in future applications.

### Strength and limits

A key strength of this study is the successful implementation of 12-session ME protocol within an inpatient setting, with high acceptability among participants. COVID-19–related recruitment barriers and inpatient TAU demands resulted in a small final sample (N = 24), substantially limiting statistical power. The number and complexity of analyses conducted increase the risk of type I error, particularly for exploratory findings. Group assignment was disclosed prior to baseline, possibly introducing expectancy or disappointment effects. Treatment integrity was not formally assessed, and interactions between participants across groups may have led to treatment contamination. Results relying solely on global self-report measures without in-session assessments may have reduced sensitivity to specific or momentary effects Further, follow-up rates were low (41%), limiting conclusions about longer-term effects. In addition, mirror exposure may elicit negative emotional responses, which should be considered when evaluating its clinical utility in this population.

## What is already known on this subject?

ME is considered a promising intervention for reducing BID in EDs, but evidence in adolescents with AN is scarce and mainly derived from multimodal treatments. Prior findings suggest that ME may affect specific behaviors such as checking or weight concerns, yet effects on global outcomes and underlying mechanisms, including attentional processes, remain unclear. This created the need to examine ME as a standalone intervention in this population.

## What this study adds?

This study suggests that ME does not provide additional benefits over focusing on weight restoration at this level of illness in improving global BID, ED pathology, or attentional bias in adolescents with AN. While exploratory findings indicate potential effects on specific behavioral aspects of BID, these results require cautious interpretation. The findings highlight the importance of established inpatient treatment approaches and suggest that the role of body image–focused interventions in this population may be limited or context-dependent.

## Supplementary Information

Below is the link to the electronic supplementary material.

Supplementary Material 1.

## Data Availability

The data that support the findings of this study are not openly available due to reasons of sensitivity and are available from the corresponding author upon request. Data are located in controlled access data storage at Freiburg University.
