# Supplementary material for: Mirror exposure in adolescents with anorexia nervosa: a feasibility study on body image disturbance and attentional bias
Source: Eat Weight Disord. 2026 Jun 8;31(1):54. doi: 10.1007/s40519-026-01880-2 (PMC13246925; doi:10.1007/s40519-026-01880-2)
Supplement: Supplementary file 1 — Supplementary Material 1. [file 40519_2026_1880_MOESM1_ESM.docx]

Supplementary Material A

*Participant Flow Chart*

*Note.* ME= Mirror Exposure group, TAU= Treatment As Usual group

Supplementary Material B

**Table B1**

*Baseline Comparison of Outcome Variables*

|  | ME | TAU |  |  |  |
| --- | --- | --- | --- | --- | --- |
|  | *n = 11* | *n = 13* | *t*(22) | *p* | Cohen’s *d* |
| FFB | 117.73 (29.65) | 144.15 (33.63) | -2.04 | .054 | 0.83 |
| BIAQ | 29.09 (11.00) | 38.92 (13.51) | -1.97 | .062 | 0.79 |
| BCQ | 56.36 (21.34) | 72.61 (16.99) | -2.04 | .054 | 0.85 |
| ChEDE-Q | 2.98 (1.03) | 3.68 (1.18) | -1.55 | .136 | 0.63 |
|  |  |  |  |  |  |

*Notes.* BCQ= Body Checking Questionnaire; BIAQ= Body Image Avoidance Questionnaire, ChEDE-Q= Child Eating Disorder Examination Questionnaire, FFB= Fragebogen zum Figurbewusstsein, ME= Mirror Exposure group, TAU= Treatment As Usual group

Supplementary Material C

**Why Did We not Find the Expected Superiority of the ME Intervention in Our Main Analyses?**

Following the unexpected finding that intensive, multi-session ME intervention did not lead to greater improvements at the BID global scale level, we conducted further post-hoc exploratory analyses at subscale and item level. This approach was based on the rationale that some of the items of the global measures may not be expected to change as a direct result of ME.

At scale level a 2 x 2 repeated measures ANOVA on the BIAQ subscale *grooming and weighing* revealed a significant Group and Time interaction (*F*(1,22) = 5.47, *p* = .029, *η*_2p_ = 0.20) as well as significant main effects of Time (*F*(1,22) = 5.47, *p* = .029, *η*_2p_ = 0.20) and Group (*F*(1,22) = 7.37, *p*= .013, *η*_2p_ = 0.25 (see Fig. S1). However, Box’s test indicated a violation of the homogeneity assumption, so these findings should be interpreted with some caution.

**Fig. S1**

*Means and Standard Deviation for BIAQ Subscale Grooming and Weighing*

*
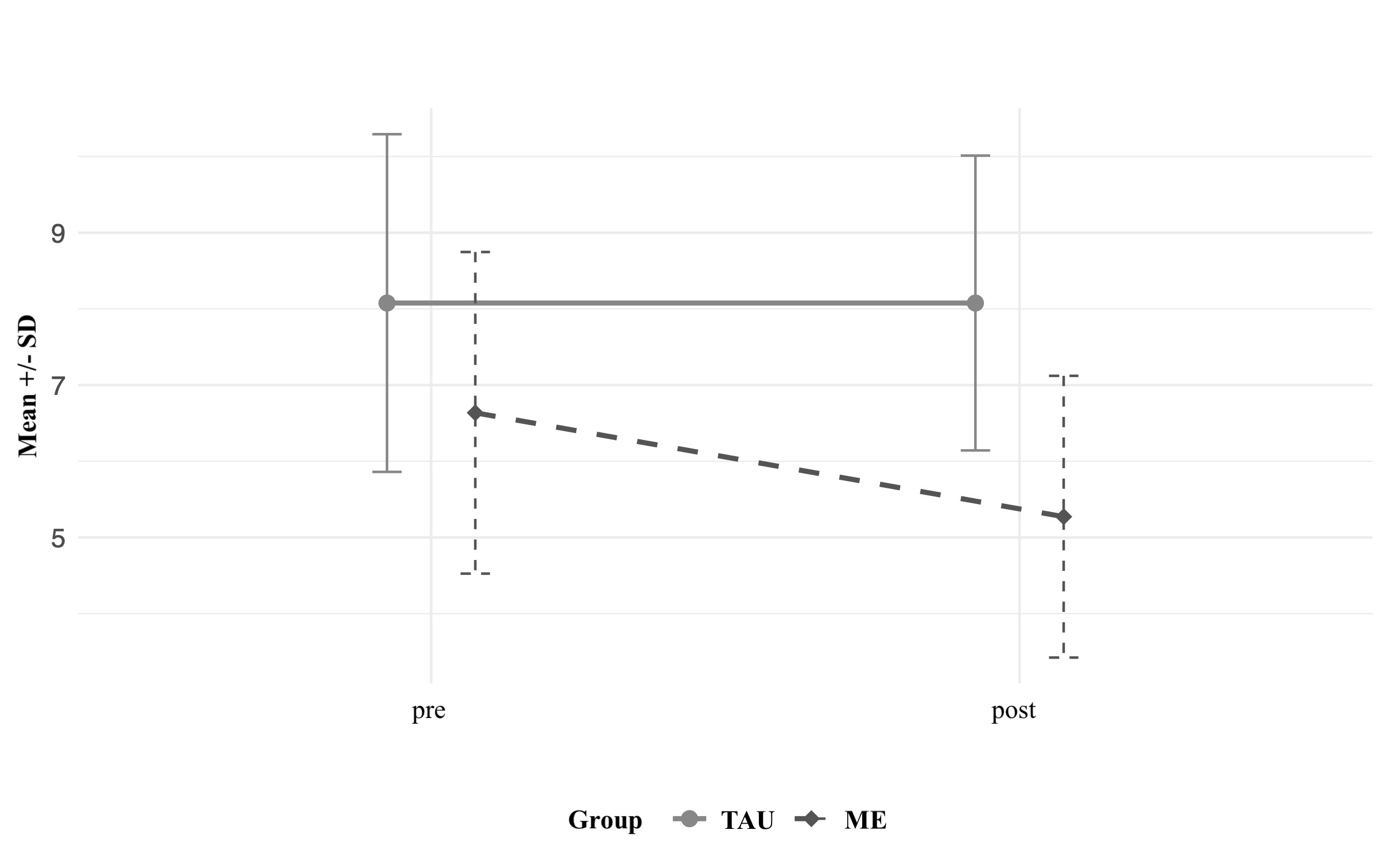
*

*Note.* Mean Score and Standard Deviation (SD) before (pre) and after (post) the intervention/waiting period for the mirror exposure group (ME) and the treatment as usual (TAU).

At the item level, several analyses revealed significant Group × Time interactions: A significant interaction was found for BIAQ item 14 (“I look at myself in the mirror”), *F*(1,22) = 8.08, *p* = .009, *η*_2p_ = .27, for BCQ item 5 (“I check my reflection in glass doors or car windows”), *F*(1,22) = 7.05, *p* = .014, *η*_2p_ = .24; and for ChEDE-Q item 24 (“How much would it have upset you if you had been asked to weigh yourself once a week...”), *F*(1,22) = 7.70, *p* = .011, *η*_2p_ = .26, see Fig. S2.

**Fig. S2**

*Means and Standard Deviation* *for BIAQ Item 14, BCQ Item 5 and ChEDE-Q Item 24*

*
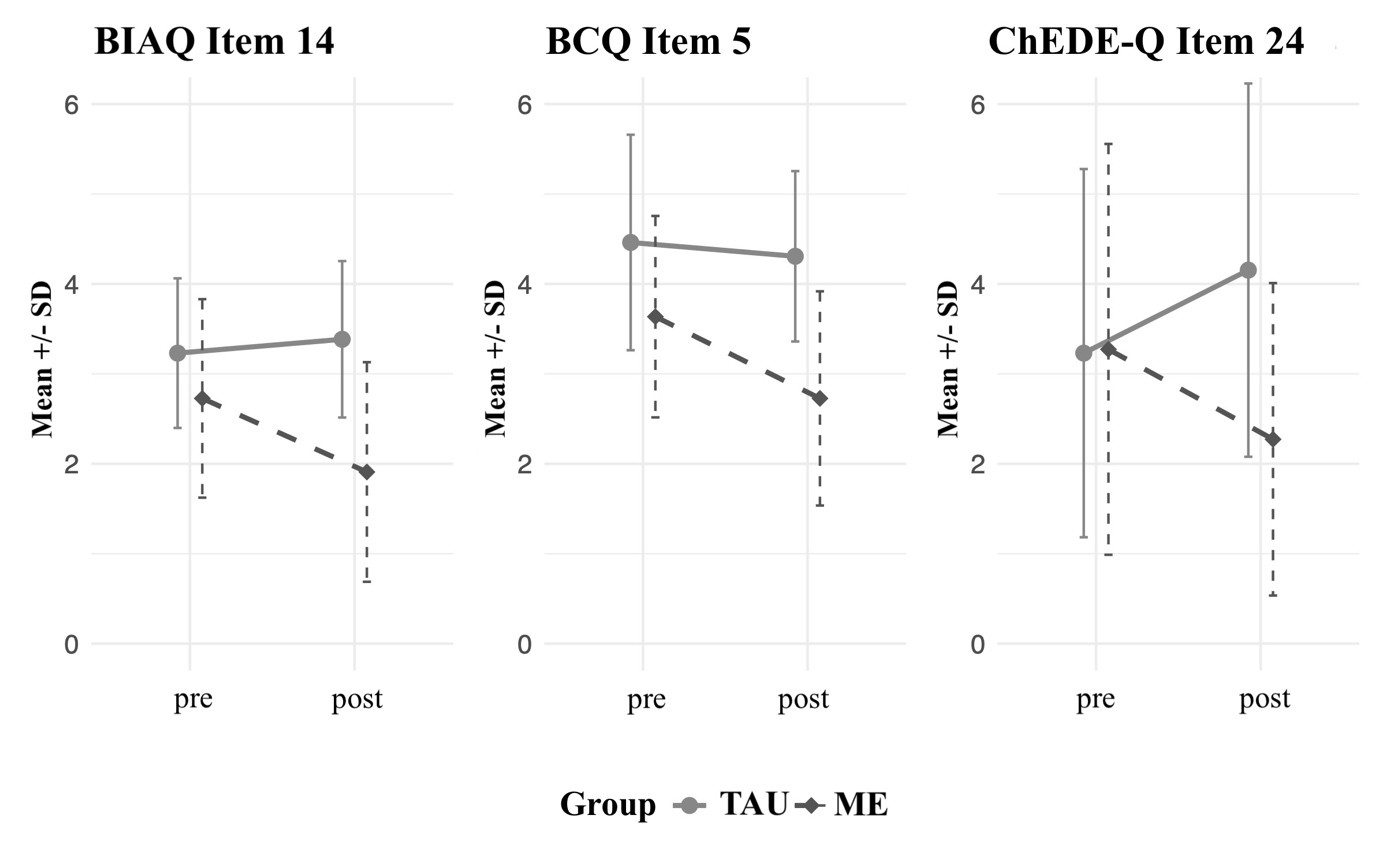
*

*Note.* Mean Score and Standard Deviation (SD) before (pre) and after (post) the intervention/waiting period for the mirror exposure group (ME) and the treatment as usual (TAU). BIAQ= Body Image Avoidance Questionnaire, BCQ= Body Checking Questionnaire, ChEDE-Q= Child Eating Disorder Examination Questionnaire.

These effects were accompanied by significant main effects of Time and/or Group, except for the ChEDE-Q item, where only the interaction reached significance. For all other scale and item-level analyses, only main effects of Time were found.
